# Symmetry recognition by pigeons: Generalized or not?

**DOI:** 10.1371/journal.pone.0187541

**Published:** 2017-11-09

**Authors:** Juan D. Delius, Julia A. M. Delius, Jennifer M. Lee

**Affiliations:** 1 Allgemeine Psychologie, Universität Konstanz, Konstanz, Germany; 2 Center for Lifespan Psychology, Max Planck Institute for Human Development, Berlin, Germany; University of New England, Australia, AUSTRALIA

## Abstract

This note looks into the reasons why earlier reports may have arrived at differing conclusions about pigeons’ capacity to categorize bilaterally symmetric and asymmetric visual patterns. Attention is drawn to pigeons’ comparatively superior visual flicker resolution and superior visual linear acuity by reporting results of two ad-hoc experiments. This circumstance turns out to constrain conclusions drawn by earlier symmetry–asymmetry studies that used computer-generated patterns displayed on cathode ray tube monitors as these suffered from pictorial distortions. Additionally one of the studies involved patterns of inconsistent symmetry at global and local levels. A smaller-scale experiment using slide-projected unequivocal symmetric and asymmetric patterns yielded results compatible with the supposition that pigeons are capable of a symmetry–asymmetry categorization. The possibility that an artfactual cue may have inadvertently accentuated this capability in an earlier own experiment is considered.

## Introduction

The research that we are about to describe has its origins with a publication by Delius and Nowak ([1]; see also [2]). That paper reported experiments indicating that pigeons were capable of categorizing symmetrical versus asymmetrical visual patterns. These experiments had employed an instrumental successive discrimination procedure in which small stimuli were back-projected on the pecking key of a conditioning chamber with the help of an automatic slide projector. The pigeons were trained with a large number of bilaterally symmetrical, and asymmetrical patterns. One group of birds was reinforced for pecking the symmetrical, and another group for pecking the asymmetrical stimuli. After having successfully learned these tasks, the same pigeons were tested in unreinforced trials with novel symmetric and asymmetric stimuli. In these tests they preferentially chose between the stimuli in agreement with their previous training. In a further experiment pigeons found it easier to discriminate between sets of stimuli that were consistently bilaterally symmetric or asymmetric than when they were not so segregated, i.e., mixed sets. At the time we concluded that pigeons were capable of categorizing visual patterns according to their mirror symmetry.

Some time later the senior author (J.D.D.) attempted to extend the symmetry research, applying a new technology and a modified procedure (Delius & Lohmann, 1985, unpublished study). The hardware was based on a Commodore home-computer, furnished with an interface and programmed with an expanded version of BASIC, a forerunner of the PSYCHOBASIC language [3]. Stimuli were presented on a cathode ray tube monitor with a black and white screen (phosphor P4, 20 cm diagonal). On any given trial it displayed an array of 12 white-on-black background pattern stimuli located 0.4 cm behind a 14.5 cm × 11 cm, 4 × 3 ensemble of transparent pecking keys. Two groups of pigeons participated in a discrete trial conditioning procedure. They were trained to choose the odd-one-out, i.e., to peck the shape displayed behind a randomly chosen key that differed from 11 other identically shaped stimuli shown behind the remaining keys. From trial to trial the patterns would be quasi-randomly selected out of a pool of 15 stimuli that all showed vertical axis symmetry and a pool of 15 asymmetric stimuli. The stimuli based on an 8 × 8 pixel field measured 5 mm × 5 mm. For one group of pigeons, the single odd pattern was always symmetric and the 11 identical stimuli were asymmetric; for the other group of pigeons the pattern allocation was the reverse (odd = asymmetric, same = symmetric). Correction trials were enforced after error choices but these were excluded from the percent correct performance score. Daily sessions consisted of 50 non-correction trials. The probability of choosing the correct key by chance was 8.3%. The 8 pigeons reached a criterion of 2 successive sessions with a score of 15% correct or better in an average 18 sessions (range 15–23 sessions). Five further sessions did not lead to any appreciable performance improvement.

Thus far the task did not require a discrimination between the symmetric and asymmetric patterns, it could simply be solved by recognizing the oddity of the one stimulus from the identity of the 11 remaining stimuli (odd–same discrimination). During the next phase of the experiment this was no longer so: on any given trial of the first group of pigeons, the one ‘odd’ stimulus continued to be a symmetric pattern but the 11 ‘same’ stimuli were in fact all different asymmetric patterns; for the second group of pigeons the symmetry–asymmetry allocation was the reverse. Disappointingly however, the pigeons had major difficulties with these tasks. After 20 sessions none of the 8 pigeons had reached the set criterion of 15% correct choices in 2 consecutive sessions. The best result we ever obtained was from one pigeon that achieved 14% correct choices on a single session. The average performance across all 8 pigeons on session 20 was barely above chance at 9% correct choices on average (range 6%–11%).

We inferred that the task was too complex for pigeons to master and abandoned it without attempting any of the further steps originally envisioned: transfer to novel symmetric and asymmetric stimuli, to stimuli with horizontal-axis symmetry, and so forth. We did, however, go on using the initial phase of the procedure to explore certain aspects of the same–different discrimination capabilities of pigeons, but without ever obtaining a really satisfactory performance level (Delius & Friesel, 1988, unpublished experiments; but compare [4]). A triple-stimulus oddity-from-sample procedure using a slide projector yielded markedly better results [5,6] (see also [7]).

The topic of symmetry–asymmetry recognition by pigeons re-attained actuality some years later when Huber et al. [8] reported that they had not been able to replicate the Delius and Nowak [1] results using predominantly a computer monitor technique to present the stimuli. Prompted by this report, and by a specific critique to be reported at the end of the General Discussion section, the senior author decided to reexamine the issue in 2002: see Experiment III. However, having been previously sensitized both to the superior flicker sensitivity and to the outstanding near vision of pigeons while preparing a review paper [9], the apparatus used in our own unsuccessful symmetry discrimination experiment (Delius & Lohmann, 1985, unpublished observations) was re-examined. Having been found wanting in two respects, this led to the two collateral Experiments I and II about stimulus flicker and stimulus degradation.

The pigeons serving were bred by Konstanz University's animal facilities. All experimental procedures employed received prior approval according to the German Tierschutzgesetz by the district's Tierschutzkommission, Freiburg-im-Breisgau, Germany. The experiments were completed before 2004, that is, just before the closure of the senior author’s animal laboratory at Konstanz University. Because the human subjects merely acted as observers of generally available light sources for human use within their standard operating characteristics no ethics issues needing any separate approval were involved according to extant German laws. Although a first-draft manuscript was completed soon afterwards, the preparation of the final manuscript was delayed until recently due to various adverse circumstances.

## Experiment I: Flicker discrimination

The reexamination of the equipment used in an earlier unsuccessful symmetry recognition experiment revealed that its stimulus display was subject to a marked flicker. This was caused by the low-frequency, 30 Hz refresh rate of the graphics generator of the computer employed. The standard refresh rate of the better computer graphics at the time of revision (2002) was meanwhile up to 50 Hz but earlier studies suggested that pigeons might even be sensitive to this higher flicker rate [10–12]. The computer graphics employed by Huber et al. [8] would almost certainly not have exhibited a higher refresh rate than that. We carried out the present experiment to ascertain whether stimulus flicker was a factor that had to be kept in mind in the context of computer-generated visual stimuli.

### Methods

#### Subjects

Six adult pigeons (*Columba livia*) of local homing stock served in this experiment, though not all pigeons were used in all phases of the experiment. They were housed in individual cages (40 × 40 × 45 cm) in a well-ventilated room that was kept on a 12–12 light–dark cycle and were maintained at 85% of their free-feeding weights. Water was freely available during the experimental sessions.

#### Apparatus

**Fig 1:**
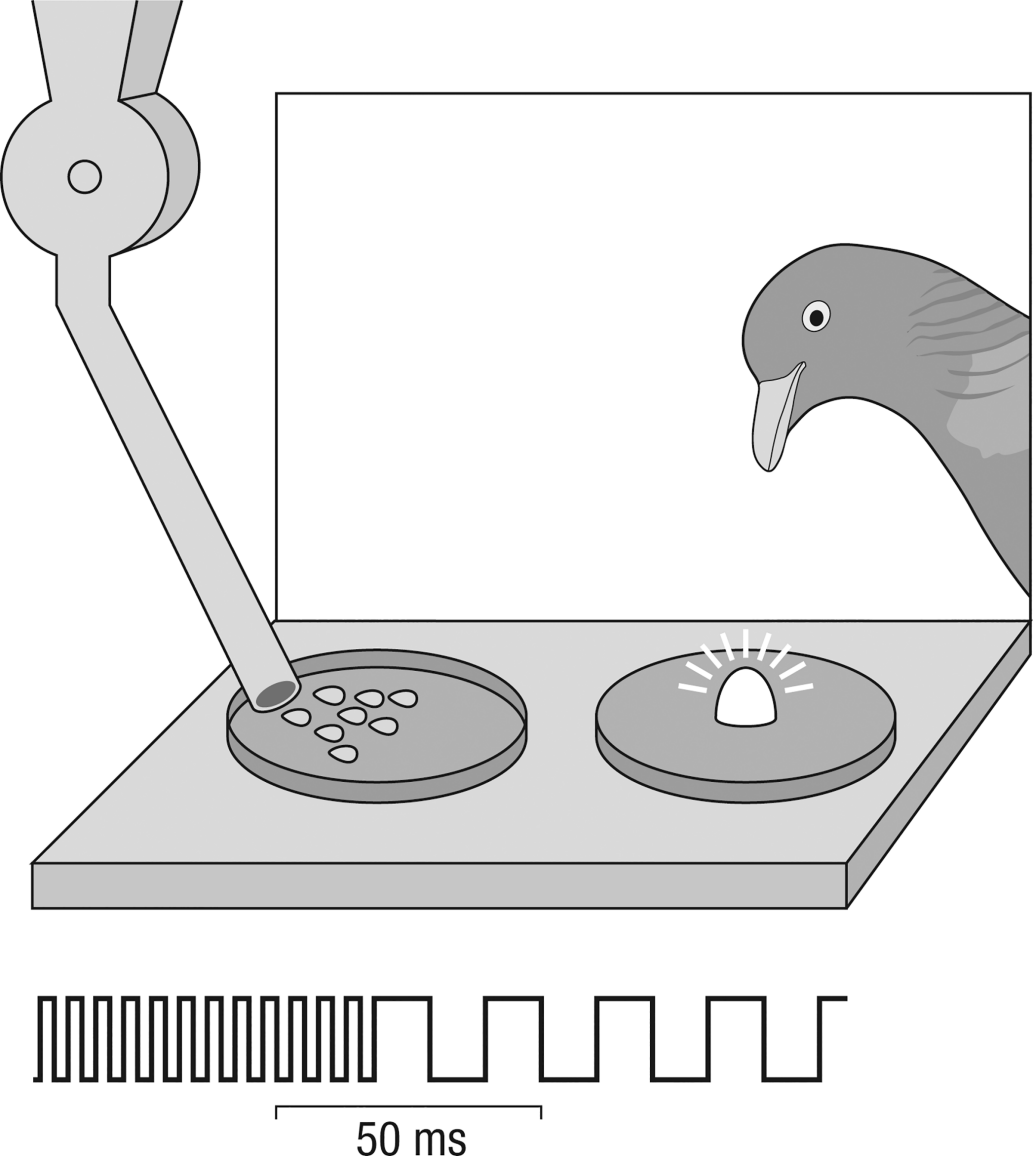
Experiment I. Flicker discrimination apparatus and S+ 200 Hz ‘steady’ followed by S- 30 Hz ‘flicker’ stimulus pulse trains driving the light-emitting diode on the impact-sensitive capsule.

We attached a horizontal conditioning platform (10 cm wide × 5 cm deep × 1 cm thick, solid steel) to the standard home cages of the various pigeons. The subjects had access to it through a wall opening in the cage that normally gave access to the food trough (Fig 1). During the experimental sessions, the platform was indirectly lit through a window with ~300 lux daylight through a photometer controlled blind. A solenoid-operated dispenser [13], affixed 15 cm above the platform, delivered three to six millet seeds through tubing into a 3 cm diameter receptacle glued onto one side of the platform. A 2.5 cm diameter impact-sensitive capsule was glued on the other side of the platform with its center 4 cm away from that of the capsule; a 2.5 cm diameter piezo buzzer was attached to the underside of the platform. Affixed on top of the capsule was a 2 mm thick white plastic disc in the center of which was a 3 mm diameter light-emitting diode. The disc-plus-diode unit was interchangeable so that in successive experimental phases we employed a green (565 nm), a red (660 nm), an infrared (810 nm), a deeper infrared (880 nm), a blue (480 nm), and an ultraviolet (375 nm) light-emitting diode. The diodes were connected through a 100 Ω resistor to a precision adjustable gain amplifier. It was used to set the on-state radiances of the diodes according to their respective voltage-radiance calibrations (see Acknowledgments and later specifications). The amplifier was supplied by one or the other of two voltage-gated precision square-wave, adjustable pulse-width, voltage-controlled frequency oscillators. The voltage outputs of the amplifier were monitored with a calibrated oscilloscope. A personal computer with interface cards served to program the stimulus presentations and to register the pecking responses of the subjects.

#### Pre-training

The pigeons were auto-shaped to peck the green 565 nm diode. Six second periods during which it was lit alternated with 20 s intervals when it was unlit. Two seconds before the lit periods ended, a reward was automatically delivered; if the bird pecked during the first 4 s of the lit periods, the reward was delivered immediately. Pecks during the unlit intervals had no consequence. A session consisted of 50 such lit–unlit cycles and there were 2 sessions per day. As soon as the pigeons began to peck regularly, the automatic rewards were discontinued and the lit periods were reduced to 4 s. The number of pecks necessary for a reward delivery was gradually increased from one to eight over several sessions. Moreover, while the former lit periods were converted into S+ periods that, at 200 Hz, would certainly be perceived by pigeons as non-flickering [11,12], the previously unlit periods were turned into lit, S- periods that flickered at 30 Hz. Both S+ and S- periods were reduced stepwise to 4 s in duration, interposing 1 s unlit intervals between the successive lit trials. The 1-to-1 on–off flickering of both S+ and S- ensured that they were of equal mean radiance. Next the sequencing of the 25 S+ and the 25 S+ trials was quasi-randomized [14]. Each peck issued during an S- period now generated a 50 ms click sound; furthermore these pecks prevented the S- period from terminating before 4 s had elapsed without a peck. Over successive sessions the reinforcement ratio was modified until it was a variable one of three pecks on average (VR3), with a range of one to five pecks. Pecks during positive and negative stimulus periods were separately counted. When 90% or more of the pecks within a session occurred during the S+ periods the birds were deemed ready for the next stage.

#### Flicker fusion determinations

Using four of the pigeons that had undergone this pre-training we determined their flicker fusion frequency employing a green 565 nm light-emitting diode set at a light intensity of 3 μW/sr. It was chosen to yield an about equal luminance of 2 log cd/m^2^ as the monitor-displayed patterns in the experiment mentioned above. The session now consisted of 2 runs of 12 blocks of randomly sequenced 100 S+ and 100 S- trials, all of these separated by 1 s intervals. There were two such sessions per day except weekends. Using a similar platform, Xia et al. [13] had found that the visual discrimination learning of pigeons was not adversely affected by a similarly massed trial conditioning procedure. During S+ trials the diode was always driven with 200 Hz pulse trains. Within each staircase run, starting from S- 40 Hz, the frequency of the S- was increased by a 5 Hz step after every 4 blocks of trials until the last 4 blocks of the trials had a 100 Hz S-. One hundred quasi-randomly selected trials were training trials as described earlier, with pecks during S+ being VR2 rewarded and pecks during S- periods trials being penalized with a click and an extension of the stimulus period. The remaining 100 trials of the block were probe trials in which pecks during the first 2 s were separately counted as correct (S+) or incorrect (S-) but had no other consequences while pecks were reinforced as above within the remaining 2 s. A criterion of at least 150 probe pecks per run was enforced: when occasionally a pigeon did not fulfill it, the run was repeated. After 2.5 to maximally 4 sessions per pigeon we had collected 5 runs that conformed to this criterion for each of the 4 pigeons.

Using the same procedure we next proceeded to determine the flicker fusion frequency corresponding to the red 660 nm light-emitting diode set to yield 100 μW/sr. The on-state radiances of this and the other light-emitting diodes to be mentioned was adjusted to yield an about equal pigeon-subjective brightness by referring to the pigeon’s known photopic spectral sensitivity [15]. However, only three pigeons were used in this instance, and only four runs that met the 150-peck criterion were exacted, these demanding 2 to 2.5 sessions per pigeon. Next we did the same using the blue 470 nm diode set to yield 100 μW/sr, and the same three birds. Three sessions were required for each pigeon to meet the minimum-response criterion. In this instance the S- ascended in 7 steps of 5 nm from 30 Hz to 60 Hz.

After completing the earlier fusion frequency determination with the green 565 nm diode, we implemented three supplementary sessions with two of the pigeons. These sessions each consisted of two runs, abbreviated to five blocks of trials going from S- 75 Hz in steps of 5 Hz to 95 Hz, while the S+ continued to be 200 Hz. This examined whether overtraining would improve the discriminative performance threshold levels. Two other pigeons were first run for three additional sessions with the stimuli S- 65 Hz (fixed) and S+ 200 Hz, both set to an 1-to-4 on–off pulse ratio with an on-state radiance intensity increased to 12 μW/sr. The latter adjustment ensured that the mean luminous intensity of the stimuli was about equal to that of the 1-to-1 on–off ratio stimuli used before. Finally, after having determined the frequency fusion of the red 660 nm diode (see above), two pigeons underwent two supplementary sessions with the diode’s radiance increased to 820 μW/sr, and with frequencies set at S+ 200 Hz and S- 60 Hz, both with a 1-to-1 on–off pulse ratio.

#### Ultraviolet and infrared flicker

Though not particularly relevant to the monitor display of stimuli issue occupying us here, these wavelength ranges are otherwise quite relevant to pigeons [15]. The peck transducer was accordingly furnished first with a near-infrared 810 nm light-emitting diode with the on-state radiance set to 6 mW/sr. Two pigeons first underwent pre-training sessions with S- 20 Hz and S+ 200 Hz stimuli. When they achieved the criterion of 90% pecks correct in that stimulus condition they proceeded to three testing runs fashioned as before but involving seven ascending S- 5 Hz steps from 35 Hz to 70 Hz. The same two pigeons were then trained to the same criterion using a 880 nm diode set at 10 mW/sr and tested in three runs between 15 Hz and 50 Hz, ascending in 5 Hz steps. Finally the same two birds were tested with the ultraviolet 375 nm light-emitting diode with its on-state radiance set to 1 mW/sr. There were five runs with seven S+ steps from 30 Hz and 65 Hz.

#### Humans

Three adult human subjects of normal or corrected vision served as observers. An abbreviated procedure was used to determine their fusion frequencies when viewing the light emitted by the various diodes set at the same on-state radiances as used with the pigeons and at reading distance (~25 cm). In four successively ascending and descending staircase runs, between 30 Hz and 80 Hz at 5 Hz per step they were required to intermittently report whether the stimulus flickered (‘yes’), perhaps flickered (‘maybe’), or did not flicker (‘no’). Having thus determined their approximate fusion frequency they were asked to set their ‘just yes’ (= fusion) frequency by adjusting the frequency setting knob of the oscillator themselves. All this could not usefully be done with the 375 nm ultraviolet and the 810 nm infrared diodes because their light was at most only visible as a very dim violet or red glow to humans. With the well visible diodes, human flicker sensitivity could by the way be much incremented by reducing the ambient lighting and recurring to repeated off-diode fixation saccades (formation of afterimage ‘ribbons’: [16]). Whether pigeons could perhaps have analogously benefited from comparable afterimages and head and eye saccades [17,18] we do not know.

### Results and discussion

For each of the pigeons a 75% correct-choice discrimination level was taken as defining the flicker/no flicker threshold (= critical fusion frequency). For each light-emitting diode and each pigeon, the mean percent correct responses were calculated for each frequency step around that threshold across the three, four, or five runs available. The 75% level intercept of an interpolated straight line was taken to be an estimate of its fusion frequency. We calculated averages across the two, three, or four relevant pigeons. Table 1 assembles these results. By the way, the sigmoid section of pigeons’ response function usually stretched over an about 30 to 40 Hz span. The humans exhibited markedly lower flicker fusion frequencies but their psychophysical sigmoid ranged over only about 15 Hz.

**Table 1:** Pigeon flicker fusion frequencies (at 75% correct choices) associated with different peak wavelength light-emitting diodes and average flicker fusion frequencies of three human observers.

Peak wavelength	810 nm	660 nm	565 nm	470 nm	375 nm
**Pigeons × runs**	2 pig. × 3 runs	3 pig. × 4 runs	4 pig. × 5 runs	3 pig. × 5 runs	2 pig. × 5 runs
**Fusion frequency**	38 (34–42) Hz	59 (57–63) Hz	73 (70–74) Hz	52 (48–57) Hz	63 (58–65) Hz
**Humans’ averages**	-------	45 Hz	44 Hz	25 Hz	-------

The green, 565 nm light-emitting diode was one of a wide spectrum type, its peak wavelength and -band approximately coinciding with the known main peak and width of the photopic sensitivity of the pigeon [15]. The overtraining to which we exposed two pigeons raised their average fusion frequencies for green 565 nm light up to 78 Hz and 81 Hz. An increased pulse on–off ratio promptly increased the mean frequency thresholds of two pigeons to 78 Hz and 80 Hz at the same wavelength; cf. [19].

As has been previously reported for birds tested at a variety of wavelengths [11,20], an intensity increment improved the detection of 60 Hz flicker of the red, 660 nm diode light by two pigeons up to a level of 78% and 83% correct choices. We prefer to consider our results concerning the near-infrared 810 nm diode as preliminary since, unlike the other findings we report, they were affected by considerable between-run variability: the preparatory training with this wavelength may have been insufficient. This applies even more pronouncedly to a few measurements obtained using an 880 nm diode on two pigeons that indicated a flicker fusion threshold at about 16 Hz. Nevertheless, as to the existence of a sensitivity to infrared light, we have repeatedly observed pigeons to be undoubtedly peck-responsive to lit infrared 880 nm diodes of photo-electric gate devices pretty much invisible to humans (J. D. Delius, unpublished observations; J. Ostheim, personal communication) but this issue clearly requires further research.

The blue 470 nm light-emitting diode again yielded a higher flicker fusion frequency in pigeons than in humans (52 Hz versus 25 Hz). A secondary near-ultraviolet photopic sensitivity peak is well established for pigeons and other birds [15,21]. Testing two pigeons with an ultraviolet 375 nm diode revealed a remarkably high average fusion frequency of 63 Hz. Even higher ultraviolet fusion frequencies have, however, been obtained with chickens [20,22,23].

In humans, flickering light undoubtedly impairs visual pattern recognition and evaluation [24,25]; in chickens, flickering lighting is widely recognized to be a visual stressor [26,27]. We do not know of a relevant published report concerning pigeons but some unpublished data obtained by Delius and Krug in 2002 is suggestive. First, four pigeons learned to concurrently discriminate six pairs of different steadily lit patterns, produced with 5 × 7 miniature under-key, light-emitting diode matrices, to a criterion of 80% correct choices. Patterns were presented behind two pecking keys on a platform described by Xia et al. [13]. When the diode matrices were switched to a 30 Hz supply, the pigeons’ performance dropped to a close-to-chance level and only recovered to an average asymptotic level of nearly 70% correct after five sessions of further training. The recognition by pigeons of the symmetry/asymmetry of visual patterns displayed on cathode ray tube displays could thus be adversely affected when graphics systems are used that are designed to surpass the flicker fusion thresholds of humans but not those of pigeons (Delius & Lohmann, 1985, unpublished observations, see above; [8]).

## Experiment II: Reversed pattern discrimination

By banking on the undoubtedly similar visuo-angular acuity of pigeons and humans [28], one tends to overlook that the superior accommodative ocular power of the former [29–31] can provide them with a markedly better visuo-linear image resolution than that achieved by humans without optical aids. It is a fact that pigeons habitually fixate grains on a substrate and stimuli on a key with eye–item distances as small as 40 mm [32–34] (but see [35]). They may go on acquiring visual information right until grasping or impacting the target thanks to a depth of focus increase due to a pupillary aperture reduction. This is brought about by a squinting of the eyelids [36] rather than a closure as asserted by Delius [37]. Emulation, with the aid of an 8× magnifying lens, of the way pigeons view a stimulus prior to pecking revealed that the vertically mirror-symmetric patterns generated by the equipment used by Delius and Lohman (1985, unpublished observations; see Introduction) were in fact subtly but consistently asymmetric. This was still the case even when examining symmetric patterns generated by, and displayed with personal computers and cathode ray tube monitors that were up-to-date at the time (2002). This must thus have affected the vertical-axis symmetric stimuli used in Huber et al.’s symmetry–asymmetry study [8]. The flaw due to the standardized left-to-right sweeping mode of the cathode ray of monitors is well known to television technicians. It was now the concern of our Experiment II, where pigeons were expected to discriminate the same square drawn on a monitor in the standard way and drawn in the reverse, right-to-left way, thus displaying different mirror symmetric flaws in each case.

### Methods

#### Subjects

Four pigeons of homing stock kept and treated as in Experiment I were used but no water was available to them during the briefer experimental sessions.

#### Apparatus

**Fig 2:**
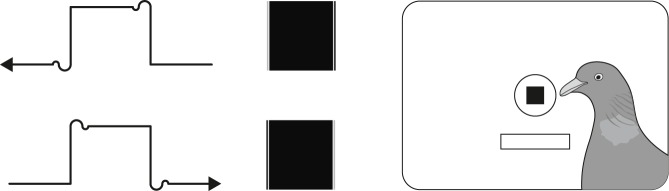
Experiment II. Right-to-left (modified, top) or left-to-right (standard, bottom) sweeping cathode ray z (brightness) signals that generated the white-on-black square patterns–shown here as negatives thereof–displayed on the monitor screen to be discriminated by pigeons.

A plywood chamber measuring 32 cm × 26 cm × 26 cm, was equipped with a 2.5 cm diameter transparent key centered 15 cm above the grid floor and recessed 1.5 cm into the back wall. The front wall and roof were made of wire grid. A solenoid feeder fixed outside delivered 6 to 9 millet seeds per activation through a plastic tube to an inside receptacle of 5 cm × 4 cm × 1.5 cm affixed 8 cm below the key. A frosted miniature 5 W house-light placed 6 cm above the key served to illuminate the chamber but no direct light from it fell upon the recessed pecking key. The same monitor (black–white, P4 phosphor) as used by Delius and Lohmann (1985, unpublished observations) was placed so that the center of its screen was located 0.5 cm behind the key. The same home computer used by these experimenters was also employed to generate a light square of 8 × 8 pixels, 5 mm × 5 mm, on a dark background precisely centered behind the pecking key using the monitor’s x-axis and y-axis adjustment potentiometers. This square pattern could be generated either with a left-to-right (= conventional) writing cathode tube beam or alternatively with a right-to-left (= unconventional) writing beam. The writing direction reversal was achieved with a switch-over, two-pole, sound-proofed relay that inverted the supply polarities to the horizontally deflecting cathode tube solenoids. Examined with a 8× magnifying lens–see above–, the normally displayed square revealed a left border light line shading and a right border dark line shading whereas the reverse displayed square exhibited a left border dark line shading and right border light line shading: see Fig 2. Though both were supposed to be identically symmetrical, the square shapes were in reality dissimilarly asymmetric. This was due to the high-frequency response limitations–resulting from unavoidable inductances and capacitances–of the brightness (= intensity, ‘z-axis’) controlling circuit of the cathode ray causing a so-called signal ‘ringing’ at the stimulus borders. Naturally the artifacts affected all vertical symmetric, as well as all asymmetric patterns displayed, but whereas they broke up the symmetry of the former they only added to the asymmetry of the latter. A noisy (‘chattering’) alternating current solenoid driven vane was interposed between the monitor face and the pecking key for 4 s during all inter-trial intervals. This black shutter prevented the bird from seeing the momentarily erratic monitor display caused by the switchover and to drown the faint switch-relay sound. A personal computer equipped with a digital interface card was programmed to control and record all the experimental states and events.

#### Pre-training procedures

The pigeons were shaped to peck the key displaying the S+ square patterns detailed below. For this they were exposed to a succession of schedules adapted from those used for a similar purpose in Experiment I. Each of the birds underwent 10 to 13 such sessions. During the last three sessions at least, the schedule incorporated a discrimination training between S+ and S-. For two birds the S+ was the left–right generated square and the S- the right–left generated square; for the other two birds the allocation was the reverse.

#### Training and testing procedures

Sessions now consisted of 30 S+ trials and 30 S- quasi-randomly sequenced trials [14]. The individual trials were separated by 4 s shutter closure and any pecks to the key during these inter-trial intervals led to a reset of the 4 s timer. Whenever required, according to the quasi-random sequencing, the switchover relay was activated or deactivated 1 s after the shutter had been operated. S+ trials lasted 25 s, of which the first 6 s (probe periods, on 20 random trials out of the 30) merely served to count pecks. Once the probe period was over, a grain reward was given with 0.5 probability if at least two pecks had been issued during the probe period. After the probe period, or right from the beginning of the other 10 probe-less trials, any pecks were reinforced on a VR10 schedule, i.e., 10 pecks on average, with a range of 5 to 15, led to reward. S- trials lasted a minimum of 10 s, of which again the first 6 s (probe periods, 20 random trials out of the 30) merely served to count pecks. Once the probe period was over, if two or more than pecks had accumulated during the probe period, there was 1 s time out (house-light off and a beep noise on). After the probe period, or right from the beginning of the other 10 probe-less trials, any pecks were punished on a VR 10 schedule by yielding a 1 s time out and initiating an at least 10 s continuation of the S- trial. This latter condition implied that from the 15th s onwards, peck responses led to a lengthening of the S- trial, thus instituting an extinction-ensuring condition. The discrimination performance was assessed solely on the basis of pecks issued during the probe periods.

### Results and discussion

Observations revealed that all four pigeons habitually fixated the key from about 8 cm distance before pecking serially. Table 2 summarizes the test performance of the individual pigeons across all their training/testing sessions. They were trained/tested until each achieved a pre-set criterion of at least five sessions of more S+ probe pecks than S- probe pecks out of six consecutive sessions. Note that the two stimuli were mirror symmetric to each other about the vertical axis (cf. [38]). This was achieved within a total of 48 correct sessions out of the total of 59 sessions across all four pigeons (binomial test, *p* < 0.001), the ratios of three of the birds being significant at the *p* < 0.05 level. The average probe peck totals and the average percent correct pecks thereof refer to the final criterion six sessions of each pigeon. A slightly rising trend in the non-tabulated session-by-session performance indicated that the pigeons’ performance had perhaps not quite reached asymptote when the experiment ended. Note though that the 30 Hz flicker affecting the stimulus displays may have impeded a more pronounced improvement (see Experiment 1). Regardless, it now seems quite likely that the artifactual asymmetry of symmetric stimuli might have interfered with the intentions of Delius and Lohmann’s (1985, unpublished) symmetry–asymmetry discrimination experiments. By extension, it may also have negatively affected Huber et al.’s symmetry–asymmetry experiments [8].

**Table 2:** Discrimination by four pigeons of squares displayed by a right-to-left (R) or a left-to-right (L) writing cathode ray monitor. It shows the average total pecks and the average correct pecks issued during the critical probe periods of all six final criterion sessions.

Pigeon, L+ or R+	#46, R+	#24, L+	#22, L+	#41, R+	Total
**Correct sessions / total sessions**	9/15	14/18	10/12	15/21	**48/59**
**Average probe pecks / criterion sessions**	133	176	156	174	160
**Average % correct pecks, criterion sessions**	70%	72%	77%	83%	75%

As far as we can judge many, if not all, monitors based on cathode ray tubes continue even presently (2017) to be affected to some extent by the consequences of the asymmetric left-to-right display generation. It is not apparent with the relatively long-distance viewing mode of humans, but it becomes quite patent when the close-up pre-pecking viewing style of pigeons is emulated with a magnifying lens. Even on an up-to-date (2017) liquid crystal monitor, symmetric shapes display fainter traces of what could be related distortions of intended vertical axis symmetric patterns. We notice that horizontal axis symmetric patterns displayed on both cathode ray and liquid crystal monitors are nearly devoid of any comparable asymmetric distortion. This could be ascertained when experimentally switching the supplies of the vertically deflecting cathode tube solenoids and instituting a non-standard bottom-to-top writing mode of our monitor’s cathode ray. Should any more symmetry–asymmetry recognition studies be carried out with computer graphics they should perhaps preferentially use the horizontal symmetry axis orientation. Separately one may still need to consider whether the locally quadruply symmetric square pixelation of modern liquid crystal monitors, although practically invisible to us, may not be visible to pigeons with their close-up viewing. The eventuality that the obligatory light polarization of these displays could disturb pigeons’ symmetry discrimination is less likely as the polarization sensitivity once claimed for pigeons [39,40] is now asserted to be artifactual, even though the evidence for the presence of such sensitivity in several other bird species is apparently growing [41].

## Experiment III: Symmetry discrimination

It is now opportune to return to the starting topic and re-examine the issue of whether pigeons can categorize the bilateral symmetry and asymmetry of planar patterns as claimed by Delius and Habers [2] and Delius and Nowak [1] but doubted by Huber et al. [8]. In view of the results of the two preceding experiments, which questioned the suitability of computer-generated stimuli in this context, we returned to the deployment of optically projected stimuli.

### Methods

#### Subjects

Three pigeons of homing stock were kept and treated as described for Experiment I except that they had no access to water during the experimental sessions.

#### Apparatus

**Fig 3:**
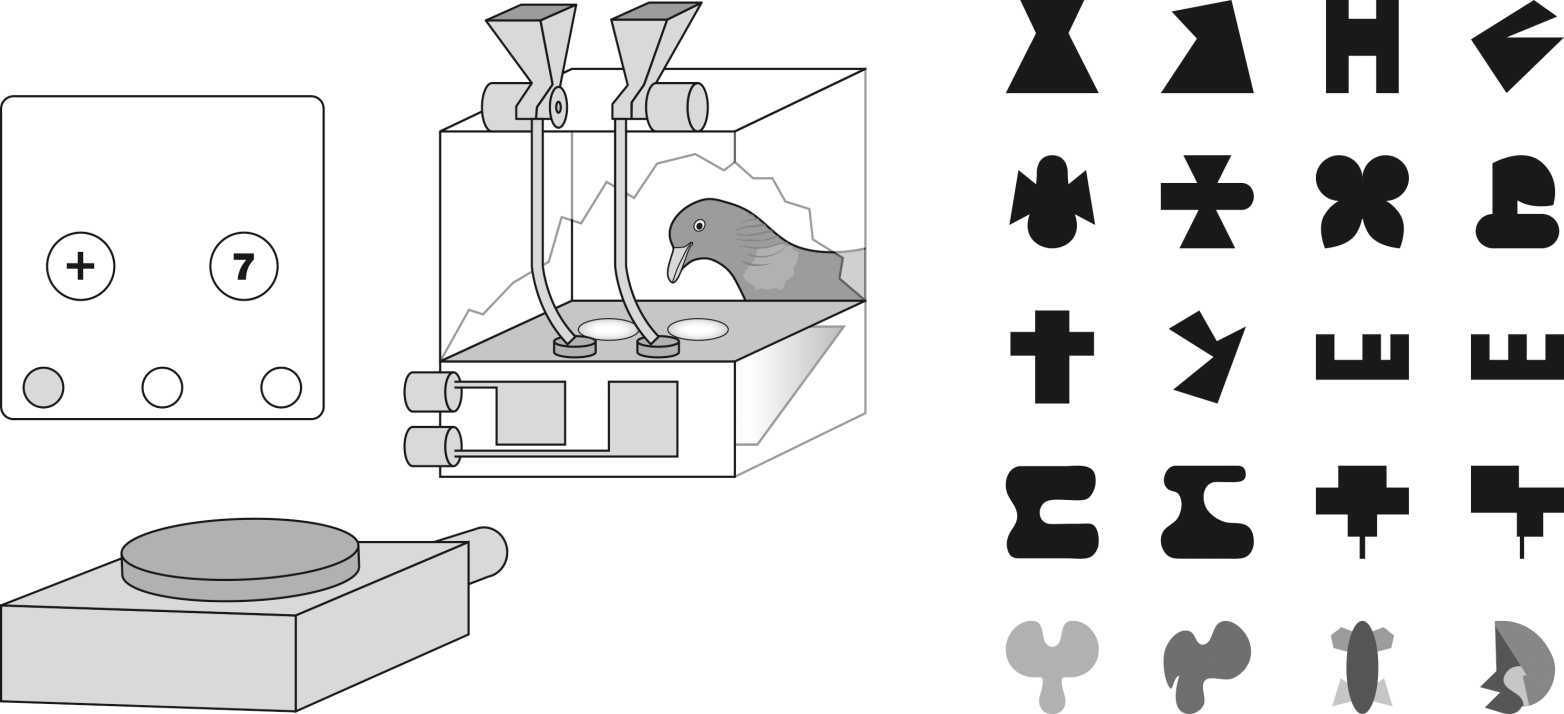
Experiment III. Apparatus with sample slide and sample symmetric and asymmetric stimulus patterns. On the pecking keys the stimuli appeared as light (or colored, bottom row) patterns on a dark background.

A 33 cm × 34 cm × 33 cm metallic chamber was employed. It had a horizontal 12 cm × 9 cm platform, 5 cm above the floor, set back as an alcove 12 cm high into the chamber’s back wall. The platform incorporated two side-by-side pecking keys, 2.2 cm in diameter, and with their centers 9 cm apart (Fig 3). Visual stimuli were back-projected onto the underside of the translucent keys with an automated slide projector via a front-silvered mirror angled at 45 degrees. The display on the keys was controlled by a pair of rotatory solenoid-driven vane-shutters. On the platform behind the keys were two circular 2 cm dishes into which two solenoid-operated food dispensers could deliver 6 to 10 millet seeds. The chamber was provided with a house-light and the alcove with a reward light [42] The projector used special slides that bore two side-by-side circular 10 mm diameter openings and three 5 mm diameter coding holes along the lower ledge. These openings corresponded to the keys and were furnished with film images of the stimuli that were fixed with transparent sticking tape. With the help of a zoom projection lens the images could be precisely centered on the platform keys. The coding holes could be occluded individually with black masking tape. Three photocells built into the projector registered the slide coding. The experiment was controlled by a personal computer furnished with an interface card and programmed in PSYCHOBASIC [3]. Note that the conditioning procedures employed here were not intended to emulate those of Delius and Nowak [1], who used a successive rather than simultaneous discrimination procedure, but were instead based on programs that had more recently proven effective in our laboratory.

#### Pre-training

Three adult pigeons of local homing stock were deprived to 85% bodyweight and were shaped to peck the keys for food reward. Incidentally, a second group of pigeons was to have been trained and tested analogously with asymmetric patterns positive and symmetric patterns negative, but this undertaking was cut short by the intervening laboratory closure (see Introduction). Twice-daily sessions were run throughout the experiment. A symmetric pattern–two different alternative symmetric stimuli being randomly used–was projected alternatingly on the right or left key for 8 s, the other key remaining dark. If the key bearing the pattern was pecked during the stimulus period, six to eight millet grains were immediately dropped into the receptacle next to it; but in any case, a millet reward was automatically issued into that same receptacle at the end of the stimulus period. The issue of rewards was invariably accompanied by a 3 s reward light onset. A pause of 20 s followed during which both keys remained dark and any key pecks yielded a 50 ms beep sound. Sessions consisted of 60 such cycles. After six such sessions the automatic rewards were discontinued, only pecks yielded reward, and the stimulus presentation gradually reduced to 5 s over the next 6 sessions.

#### Training

During discrimination training, pairs of stimuli were presented on each trial. An S+ symmetric pattern and an S- asymmetric pattern were allocated to right and left keys on a quasi-random basis [14]. The S+ and S- patterns constituting a given pair were quasi-randomly selected from those used during pre-training, which are those shown in the upper row of Fig 3. These stimuli stayed on until the pigeon pecked one of the two keys. If the key bearing the S+ pattern was pecked, a reward was delivered into the receptacle next to it, followed by a 5 s pause with no stimuli. If the key bearing S- was pecked, then this led to the beep sound and a timeout period with the house-light off for 5 s; if the dark key was pecked, this extended the timeout by another 3 s; the next trial was then a repeat correction trial. The inter-trial intervals were 5 s pauses with no stimuli displayed on the keys and there were now 80 trials per session. Any pecks at a dark key also yielded the beep sound. The reward schedule for pecks to the S+ was gradually increased to VR3 (range 1–5 pecks) while the set of patterns was increased by adding two symmetric–asymmetric pairs of patterns per session. The latter, although different from them, were in the same style as the ones shown in Fig 3 and were borrowed from Delius and Nowak (cf. Fig 1 in [1]). The increase of the pattern set continued until the pigeons were routinely dealing with a total of 24 different patterns, 12 of each kind, which were randomly rearranged into new pairs before every second session. When a pigeon completed 2 consecutive sessions with 80% or more of its pecks at the S+ symmetric patterns at this stage, it proceeded to the next stage.

#### Testing

Within each of the subsequent sessions there were 16 out of the 80 trials in which pecks had no consequence for the pigeons. Eight of these trials included the unreinforced presentation of an old symmetric–asymmetric pair of patterns (dummy tests); the other eight trials involved the unreinforced presentation of a novel symmetric–asymmetric pair of patterns (true tests). The pecks delivered during the individual trials of these two types of tests were separately counted as correct (directed at S+) or incorrect (directed at S-). Any trial that yielded more S+ than S- pecks counted as a correct trial; any trial that yielded equal numbers (only a few such trials occurred) or more S- than S+ pecks counted as an incorrect trial. Any test that yielded more correct than incorrect trials counted as correct. Any test that yielded as many (i.e., four of each) or more incorrect than correct trials, counted as incorrect. The purpose of the dummy test pairs was simply to reduce the chance that pigeons would come to associate non-reinforcement with stimulus pattern novelty, which might have depressed test pecking rates. A session always began with 10 training trials and ended with 10 training trials. The 16 test trials were inserted quasi-randomly among the remaining 44 training trials.

In a total of 34 such sessions we successively introduced one by one: (a) 8 novel test pairs, (b) another 4 such pairs, and (c) a further 4 novel test pairs. Series (c) was special insofar that it incorporated a coding reversal of all the symmetric–asymmetric stimulus slides used in the relevant sessions accompanied by corresponding computer reprogramming: the exercise was meant to control the possibility that the optical slide coding was somehow a cue that was accessible to the pigeons (see General Discussion below). It was followed by sessions involving the introduction of (d) six novel test pairs incorporating oblique and vertical axis symmetry patterns, (e) six novel test pairs of piled bar design patterns, and finally (f) six test pairs involving novel colored symmetric and asymmetric patterns. After a novel pattern had served as a test pattern during one session, it was routinely added to the set of training patterns used in subsequent sessions so that towards the end of the experiment, i.e., during the color test (f), the pigeons were dealing with a total of 52 different white-on-black symmetric and asymmetric stimuli.

### Results and discussion

No particular difficulties were experienced with training the three pigeons to discriminate a pool of 24 symmetrical from 24 asymmetric white-on-black patterns up to an 80% correct performance criterion (Table 3). Note, however, that this achievement is easily within pigeons’ capacity to learn to discriminate the patterns by rote [43,44]. But when their generalization to 28 novel symmetric and 28 novel asymmetric patterns was tested unreinforced, all three pigeons also preferred the symmetric stimuli: overall 78%, (range 67%–85%) choice. The overall ratio of 66 positive to 18 negative test results was highly significant (binomial test, *p* < 0.001), and the individual ratios of two of the three birds were significant at the *p* < 0.05, and that of one at the *p* < 0.01 level.

**Table 3:** Symmetry–asymmetry true tests. Categorization of novel stimuli by three pigeons. Bracketed figures refer to dummy tests (see text). The but-last row lists the correct tests out of the total tests (a) to (e).

Pigeon	#31	#44	#36	Total
**a) test correct/test total**	6/8	6/8	7/8	19/24
**b) test correct/test total**	3/4 (2/4)	4/4 (3/4)	3/4 (4/4)	10/12 (9/12)
**c) test recoded slides**	3/4 (4/4)	3/4 (3/4)	3/4 (3/4)	9/12 (9/12)
**d) test oblique & transversal**	4/6	6/6	6/6	16/18
**e) test bar patterns**	3/6	5/6	4/6	12/18
**correct/total tests,** **% correct**	**19/28** 67%	**24/28** 85%	**23/28** 82%	**66/84** 78%
**f) test colored patterns**	2/6	4/6	3/6	9/18

Note, however, that we excluded from this overall assessment the test (f) employing variously colored test pairs: with these the pigeons’ discrimination broke down. Nowak and Delius (unpublished data) had obtained a similar discriminative collapse when attempting to extend their 1982 study [1] to colored symmetric and asymmetric patterns. In the present case, the performance with three uniformly but diversely colored symmetric–asymmetric patterns recovered upon training with the same stimuli but remained poor with three tri-colored patterns. Initially, the pigeons’ spontaneous color preferences [45,46] may have temporarily overridden the trained symmetry preference. The performance with tri-colored pattern pairs was abysmal as the symmetric patterns tended to occasion pecking refusals that even brought about preferences for asymmetric patterns. The former patterns were possibly avoided because they were reminiscent of aposematic (warning colored) insects: local pigeon fanciers know their pigeons to be wary of wasps and bumblebees; cf. [47]. Within Nowak and Delius’ efforts, a more extended exposure to colored patterns had incidentally also transiently inhibited the previously excellent discrimination of white and black symmetric and asymmetric patterns. A sudden switch from white-on-black to colored stimuli incidentally was also found to inhibit the discriminatory performance of pigeons faced with a quite different task [48]. Note, incidentally, that pigeons’ color vision differs markedly from that of humans [9].

Tests (a) to (d) all evinced satisfactory levels of discrimination transfer to novel stimulus pairs. Note that test (c) incorporated a reversal of the slide coding so that, if the coding was a cue to the subjects, it should have led to an inversion, or at least a depression, of the trained symmetry preference, both within the true and the dummy tests. However, there was no evidence of this when the results of test (c) were compared with those of test (b) involving normally coded slides. Further we note that test (d) involved test pairs with symmetric patterns whose axis orientation was oblique or transverse from the previously standard axis. This had no detectable influence on pigeons’ performance as one would indeed expect from horizontally displayed flat patterns, where their orientation and that of the subjects was largely arbitrary [49].

Test (e) concerned symmetric and asymmetric patterns constructed out of bars. Stylistically these patterns were different from the ones we previously employed. Their design was taken from that of the ‘compact’ patterns that Huber et al. [8] had found to yield negligible symmetry–asymmetry discrimination. Except for test (f) with colored stimuli, these patterns also yielded the weakest of our test results: 66% correct choices across the four pigeons. But we must remark that after having been incorporated into the training pattern sets for two sessions, the same pattern pairs yielded an improved score of 13/18 (72% correct) when being specially dummy-tested again on a third session. Whether a style novelty, see later, and/or an insect similarity had negatively influenced the initial choice behavior of the pigeons remains uncertain.

## General discussion

As limited and constrained as they are, the results of Experiment III do nevertheless support the view that pigeons are capable of at least a partial categorization of visual patterns into symmetric and asymmetric classes. This generally agrees with the position taken by Delius and Nowak [1] but not with the conclusion reached by Huber et al. [8] after some further, very pertinent experiments with pigeons to which we already had occasion to refer. Their first experiment involved the discrimination learning of sets of symmetric and asymmetric patterns and led to a second experiment involving generalization tests with novel sets of such patterns. In an early phase their stimuli were optically projected and in a later phase monitor-displayed but the resulting data were presented pooled. There were two groups of pigeons, one trained with ‘compact’ patterns and another one trained with ‘scattered’ (i.e., spotted) patterns. The white-on-black patterns, all inscribable within 1 cm^2^, making up the compact sets were composed of six horizontal, variously elongated bars–each by itself a both vertically and horizontally symmetrical rectangle–that were symmetrically stacked around a vertical axis, or alternatively asymmetrically stacked around a vertical line. The former ‘symmetric’ patterns were thus all globally and locally symmetric whereas the latter ‘asymmetric’ patterns were all globally asymmetric but locally symmetric patterns.

The patterns composing the scattered sets were all constructed within a 9 × 9 matrix with between 12 and 18 squares, each by itself quadruply symmetric, that is simultaneously mirror symmetric about their vertical, horizontal, and diagonal axes. These squares were pairwise disposed symmetrically either around a single vertical, a single horizontal, or a single diagonal axis in nearly half of the patterns, or simultaneously around the vertical and the horizontal axes, or also simultaneously symmetrical around the vertical, the horizontal, and the diagonal axes in nearly another half of the patterns, being thus globally either mono- or multi-symmetric, and locally multi-symmetric patterns. Disconcertingly, two of the patterns, although locally multi-symmetric, were globally not mirror symmetric at all but rotation symmetric instead. Incidentally, the multi-bilaterally symmetric patterns were also additionally all point-symmetric. The asymmetric scattered patterns also consisted of 12 to 18 all quadruply symmetric squares that were globally asymmetrically arranged around any and all axes. We note here, however, that at a regional level (i.e., between local and global level), groups of squares also happened to make up many symmetric and asymmetric features in both the symmetric and asymmetric patterns. As to the more general role of local and global stimulus aspects in visual perception experiments of pigeons, see [50–52].

The pigeon group presented with ‘scattered’ (= spotted) stimuli learned to discriminate the training sets quite well up to the equivalent of an about 90% correct asymptote whereas the group presented with ‘compact’ stimuli did so poorly, reaching only the equivalent of an about 60% correct asymptote. When generalization tests were carried out in Huber et al.’s second experiment [8], the ‘scattered’ group transferred their discriminative behavior quite well to the sets of novel symmetric and asymmetric scattered patterns but transferred it very poorly to sets of novel symmetric and asymmetric compact patterns. Conversely so the ‘compact’ group, but of course to a markedly lower overall level of performance in accordance with their deficient original learning. Thus far the results could be seen as reflecting the different degrees of conjoint local and global symmetry–asymmetry explicated above.

Next, however, followed a test with novel patterns that were all derived from symmetric patterns of each training set. These were sparingly modified by changing the positions of just a few squares to yield three novel globally symmetric patterns and three novel globally asymmetric patterns. A significant majority of these patterns, regardless of whether actually symmetric or asymmetric, were classed by pigeons as symmetric, or more accurately as simply similar to the original (symmetric) pattern. Huber et al. [8] concluded that the pigeons may have generalized solely according to pattern similarity all along and not at all according to pattern symmetry. They argued that it is inappropriate to talk about anything like a symmetry categorization capability. But there is of course the difficulty of the local symmetry pervading the globally asymmetric patterns too.

In their third experiment, Huber et al. [8] ran a further three groups of pigeons, all the while displaying the stimulus patterns on a computer-driven monitor. Group A was trained and tested using a behavioral procedure like that employed by Delius and Nowak [1] with the above ‘compact’ patterns; group B was trained using the same type of procedure with patterns like those used by Delius and Nowak [1]; and group C was trained using a behavioral procedure like that originally used by Herrnstein, Loveland, and Cable [53], in fact the same as Huber et al.’s [8] experiments 1 and 2 described above, but on patterns like Delius and Nowak’s [1]. Only group C showed any proper discrimination learning, achieving an asymptote of about 70% correct choices. Upon testing with novel patterns of the type used by Delius and Nowak [1], all groups evinced a transfer performance that was at best barely above 50% correct choices. Remarkably, however, during the testing phase group C’s performance on the training component suddenly shot up to an almost 80% correct level, a fact that was not commented upon: was it due to a behavioral contrast effect, an improved performance that sometimes appears when a more difficult component is added to an easier task [54,55]? Note that in Delius and Nowak [1] and the present Experiment III, the white-on-black stimulus patterns were all–except those of test (e)–solely globally symmetric or asymmetric and all locally neutral in this respect.

Could the overall poorer performance of groups A, B, and C be due to the stimulus flicker inherent to the presumably 50 Hz refresh rate of the graphics generator used for the most part of the experiments (see our Experiment I); to the vertical axis symmetry distortion caused by the lateralized pattern generation mode of the cathode ray monitor used (see our Experiment II); or to the monitor screen displaying the small patterns 4 cm behind the key when the pigeons, needing to fixate the key from 8 cm away and closer, perhaps had to view the stimuli from 12 cm away instead, an unusually large pre-peck stimulus fixation distance [34,56]? In any case, Huber and other co-authors [57] drew attention to some of these defects later and have devised a discrimination apparatus for pigeons using a liquid crystal display and an infrared touch screen that largely avoids the drawbacks listed above. We would still query their use of a formerly meat-producing, now largely fancy breed of pigeons (Austrian strassers) rather than homing or feral pigeons whose intelligence is doubtlessly under harsher everyday selection pressure. But their newer technique, successful in other respects, has not yet been employed to re-examine the symmetry recognition issue.

In turn, we must also reconsider the devices used by Delius and Nowak [1] in their pigeon symmetry recognition study. When J.D.D. referred to the results of the study in a seminar in 2000, he was asked whether the stimulus slide coding might not have provided the pigeons with an unintended cue. An instance was mentioned where a similar, though reflective, slide coding used in a tachistoscope based on a slide projector had turned out to provide an unintended faint chromatic fleck cue to human subjects. Apart from arguing that such a cue seemed physically unlikely with our transmissive form of coding, and that the on-key stimuli had been routinely controlled for sharpness without ever noticing any such cue, J.D.D. could not fully dismiss the objection as we had not run an appropriate control, a must in view of pigeons’ sensitivity to ultraviolet and infrared light: see Experiment I. Retrospectively considered, perhaps some such unintended cueing might have brought about the rather unusually high number of scores of over 90% correct choices achieved by Delius and Nowak’s pigeons [1]. However it cannot have been the whole explanation, as the differential results of the symmetry-relevant/symmetry-irrelevant discrimination experiment within that same study cannot be attributed to the pigeons’ detection of a mere artifact. Neither can it explain the results of a later experiment using the same projector and coding that yielded negligible evidence of point symmetry generalization by pigeons (Delius & Bothe, 1984, unpublished data). Note that the present Experiment III incorporated an adequate control procedure which did not yield any hint that the slide coding might have provided a cue influencing the choices of the pigeons though now using a quite different slide projector. In any case, the earlier Delius and Habers [2] results that also supported a symmetry recognition in pigeons were obtained with inline projectors not requiring any kind of optical coding.

It remains to speculate what function a generalized symmetry recognition might fulfill for pigeons in their natural environment. Delius and Nowak [1] suspected on general grounds that selective filters for symmetry and asymmetry had to be a basic feature of all advanced visual systems. More recently Sawada, Li, and Pizlo [58] have managed to experimentally support the view that a generalized symmetry detection mechanism underlies the cognitive capacity that allows humans, and doubtlessly other advanced vertebrates, to reconstruct the three-dimensional structure of real objects out in space from the two-dimensional images these cast on their retinas. There may even be an extrastriatal cortical brain area specialized on the recognition of bilateral symmetry [59]. Nevertheless it seems likely that through evolutionary exaptation [60] such a filter might have been put to more specific uses by a variety of animals, see [61,62]. Following Menne and Curio’s [63] idea that symmetry recognition was useful for insectivorous birds like titmice (*Parus sp*.) as a camouflage-breaking device, Delius and Nowak [1] assumed that it could be useful to pigeons for the detection of grains and seeds against variegated backgrounds. This signifies that symmetry could be a gestalt factor aiding a figure-from-ground segregation [52]. But more recently others have followed up evidence that symmetry recognition plays a role for birds in partner-identification processes, [64] for chicken, *Gallus gallus*, and in mate-quality assessment, [65] for swallows, *Hirundo rustica*, [66,67] for starlings, *Sturnus vulgaris* (see also [68]). However, this context would involve larger-scale stimuli viewed from some distance rather than small stimuli viewed from nearby as in our experiments. In pigeons, who might be suitable experimental subjects as they occur with symmetric and asymmetric plumage colorations, this would implicate the lateral visual field rather than the frontal one that has occupied us here. With human subjects the mechanism of recognition of large-scale (global) symmetry is thought to differ from that subserving the identification of small-scale (local) symmetry [69].
